# Secure and decentralized federated learning framework with non-IID data based on blockchain

**DOI:** 10.1016/j.heliyon.2024.e27176

**Published:** 2024-02-29

**Authors:** Feng Zhang, Yongjing Zhang, Shan Ji, Zhaoyang Han

**Affiliations:** College of Computer Science and Technology, Nanjing University of Aeronautics and Astronautics, Nanjing, 211106, China

**Keywords:** Blockchain, Federated learning, Smart contract, Non-IID data, Privacy preservation

## Abstract

Federated learning enables the collaborative training of machine learning models across multiple organizations, eliminating the need for sharing sensitive data. Nevertheless, in practice, the data distributions among these organizations are often non-independent and identically distributed (non-IID), which poses significant challenges for traditional federated learning. To tackle this challenge, we present a hierarchical federated learning framework based on blockchain technology, which is designed to enhance the training of non-IID data., protect data privacy and security, and improve federated learning performance. The framework builds a global shared pool by constructing a blockchain system to reduce the non-IID degree of local data and improve model accuracy. In addition, we use smart contracts to distribute and collect models and design a main blockchain to store local models for federated aggregation, achieving decentralized federated learning. We train the MLP model on the MNIST dataset and the CNN model on the Fashion-MNIST and CIFAR-10 datasets to verify its feasibility and effectiveness. The experimental results show that the proposed strategy significantly improves the accuracy of decentralized federated learning on three tasks with non-IID data.

## 1 Introduction

Federated Learning [1]is a distributed machine learning approach that facilitates collaboration among multiple devices or edge devices for training machine learning models, all while preserving the privacy and security of sensitive data without the need for sharing or exposing it. In traditional centralized machine [2] learning methods, data is usually required to be centralized in a data center or cloud service for training. On the contrary, Federated Learning decentralizes model training across multiple devices, where each device solely handles the processing of its local data. This approach significantly enhances data privacy and security.

**Figure 1:**
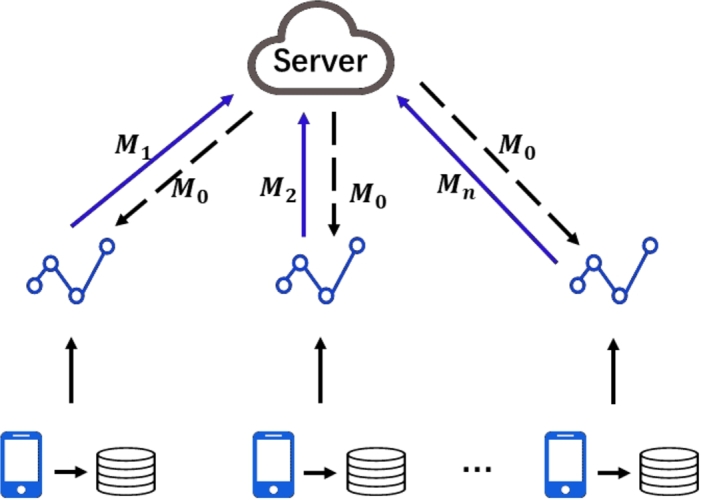
Federated Learning Framework.

The fundamental concept behind Federated Learning involves deploying the machine learning model to the participant's local device. The model is then trained on the local device to generate local updates, which are exclusively stored on the local device. These local updates are then aggregated to generate a global update, which is sent back to the centralized server [3]. The central server merges the global update with the original model and sends the result back to the participant. This process can be iterated repeatedly until the model converges. The basic framework of federated learning is illustrated in Fig. 1.

Federated Learning can be categorized into three types: horizontal Federated Learning [4], vertical Federated Learning [5], and Federated Transfer Learning [6]. The characteristics of horizontal Federated Learning are that the data features of each client are the same, while the users are different, which can be understood as an extension of the sample quantity. The distinguishing feature of vertical Federated Learning and Federated Transfer Learning is that the users remain consistent or the same across the different stages or tasks, but the data features are different, which is an extension of the sample data features. The research on addressing the non-IID data [7] distribution challenge in Federated Learning primarily concentrates on horizontal Federated Learning classification [8]. The concept of ‘same distribution’ implies that the data exhibits stable trends, with a consistent and unchanging probability distribution across all samples. Independence refers to the absence of correlation between individual samples, where each sample is considered to be independent. In traditional distributed machine learning, sub-datasets are derived from a larger dataset, and these sub-datasets can effectively represent the overall distribution. However, in the context of Federated Learning, the private data held by each device is not randomly collected or generated, leading to a certain level of correlation and violating the assumption of independence. Additionally, the local data sizes of individual devices differ, further deviating from the notion of a uniform distribution. The presence of imbalanced data distribution caused by non-IID data can introduce biases during model training and potentially result in performance degradation within the Federated Learning framework. Consequently, addressing the challenge of non-independent and non-identically distributed data has emerged as a critical research focus in the field of distributed machine learning.

In federated learning, participants typically have their own datasets and local models, but these datasets cannot be shared due to data privacy concerns. Therefore, participants need to share the parameters of their local models with other participants through encryption and other methods to achieve model updates and merging. In this process, data privacy and security face challenges. Blockchain technology [9] can ensure private data security and privacy through decentralization, trustworthiness, and encryption. On the blockchain, each participant can have their own data node and control data sharing and access through smart contracts [10]. Participants can share the parameters of their local models with other participants through blockchain technology while protecting data privacy and security.

This article presents a federated learning framework based on blockchain technology, designed specifically for training non-IID data. By utilizing smart contracts to achieve decentralized federated learning, the proposed framework overcomes the difficulty of training non-IID data and efficiently protects model security and data privacy. Due to the imbalance of local client data, a challenging problem arises: how to train non-IID data securely and effectively to coordinate the decentralized learning process while maintaining model updates and aggregation.

With this objective in mind, this article introduces a federated learning framework based on blockchain technology, specifically tailored for training non-IID data. The key contributions of this article are as follows:

• To better train non-IID data, reduce model training bias, and improve federated learning performance, we propose a hierarchical blockchain system consisting of a main blockchain for global model aggregation and a blockchain-based data sharing layer for local model aggregation. By sharing a small amount of data, we enhance model prediction accuracy.
• We utilize smart contracts to distribute the global model and receive locally aggregated models, enabling decentralized federated learning that better protects user data privacy and resists model attacks.
• We designed a model similarity calculation module, which visualizes the performance of the model by calculating the degree of dissimilarity between the local models and the initial model through a model similarity test.
• Through detailed experiments on three datasets, we demonstrate that our proposed federated learning system achieves better model accuracy and faster training efficiency than traditional federated learning models.

## 2 Related work

### 2.1 Heterogeneous federated learning

Compared to traditional machine learning, which focuses on training in centralized data centers, federated learning does not require data sharing among edge devices. This enables collaborative training while safeguarding data privacy and security. As a result, federated learning has gradually emerged as a prominent research area in machine learning [11], [12], [13]. However, despite its growing popularity, federated learning is still in its early stages of development and faces numerous challenges.

One significant concern in the field of federated learning is system heterogeneity. Heterogeneity refers to the differences among participants, including data heterogeneity, hardware heterogeneity, and network communication heterogeneity. Data heterogeneity can be further categorized into distribution, scale, and quality heterogeneity [14]. These differences can have negative impacts on the performance of federated learning, necessitating the development of appropriate optimization and adjustment strategies to address heterogeneity, such as model balancing, data selection, and aggregation rules. This paper primarily focuses on the issue of data heterogeneity.

### 2.2 Federated learning with non-IID data

With the large-scale use of the framework, federated learning will also face the problem of non-IID due to different scenarios and users, as it involves the collaborative training of models on tens of thousands of devices and their private datasets. The imbalanced distribution of non-IID data can introduce bias during model training and potentially result in a decline in the performance of federated learning. The problem of non-IID lies in the fact that the datasets of each device are usually collected from different users and different scenarios, so the assumption of IID data fails to satisfy the model's generalization capability.

To tackle the adverse effects of data heterogeneity, Wang et al. [15] addressed the divergence issue arising from the non-IID nature of distributed datasets. Li et al. [16] introduced a committee-based cross-validation approach to effectively handle non-IID datasets and update the global model. Shin et al. [17] employed a hybrid approach to enhance non-IID data by augmenting the training data's diversity, thereby mitigating the challenges associated with non-IID training. In addition, data augmentation can also be achieved through methods such as random transformation and knowledge transfer [17]. Yoshida et al. [18] proposed a new federated learning mechanism, Hybrid-FL, which designed a heuristic algorithm that allows the central server to collect data from devices with low privacy sensitivity to construct independent and identically distributed data, and used the constructed data to train the model and aggregate it into a global model. Wang et al. [19] proposed an aggregation algorithm for model selection, aiming to improve the accuracy of the aggregated global model by identifying and excluding models with biases in local updates.

Zhao et al. [8] proposed a strategy that creates a data subset shared among all private datasets to reduce the gap between each model and enhance the training accuracy of non-IID data. The idea of data sharing in this paper was inspired by this work and references [20], [21], [22]. This approach not only offers ease of implementation but also significantly boosts the performance of federated learning when dealing with non-IID data. In reference [14], sharing a fraction of the entire dataset, comprising 10%, resulted in an approximate 30% increase in model testing accuracy.

### 2.3 Blockchain in federated learning

In federated learning, the central server plays an important role in aggregating local models of edge devices. Under the security challenges, the central server faces, an unstable central server can cause system crashes. The consensus mechanism commonly used in blockchain research is Proof of Work (PoW) [23], which can effectively prevent Sybil attacks. When creating a new block of information, the algorithm generates a related cryptographic puzzle that must be solved to verify/add new records. All computer nodes (miners) in the network attempt to solve this puzzle to earn transaction fees and cryptographic rewards [24]. It is widely employed in the cryptographic community, particularly in addressing sensitive data such as financial and healthcare-related issues. PoW ensures data traceability, meaning that the complete history of any record/transaction can be traced, examined, and remains unalterable [25], [26]. When applied to the federated learning framework, the blockchain-based consensus algorithm updates and distributes the model parameters to users after cross-validation [27], [28]. Blockchain has security features such as decentralization and tamper-proofing, which can solve the security issues of the central server in federated learning. Therefore, the amalgamation of blockchain and federated learning has garnered considerable attention [29], [30].

The pioneering research on blockchain-based federated learning is the impact of block creation and consensus delays in the federated learning process [27], [31], [32], [33], and since then, blockchain has been continuously applied to various new federated learning frameworks. Kim et al. [34] introduced a federated learning framework based on blockchain, where local models are uploaded to the blockchain network following local training. The framework utilizes Fedavg for model aggregation after consensus is reached by miner nodes, and provides rewards to devices based on output rate parameters. Li et al. [35] presented a blockchain-based federated learning framework called BFLC, which extensively addressed the model storage method, redesigned the training process, and introduced a novel consensus mechanism. Desai et al. [36] implemented and evaluated blockchain-based federated learning using Hyperledger Fabric. Qi et al. [37] proposed a federated learning model based on a homomorphic integration blockchain, which uses homomorphic encryption for gradient privacy protection, and uses an on-chain/off-chain storage strategy and smart contract-based reputation scheme to address federated learning trust and blockchain storage issues. Cao et al. [38] proposed the DAG-FL architecture, which uses the DAG blockchain architecture to improve the model validation mechanism of decentralized federated learning, effectively detects abnormal nodes, and proves its effectiveness through experiments. Zhou et al. [39] proposed a secure distributed machine learning framework based on blockchain and a 5G network, which solved the availability problem of federated learning with the communication speed of a 5G network. They also introduced blockchain sharding technology and designed a set of gradient anomaly detection strategies. Jin et al. [31] conducted groundbreaking research on blockchain-based federated learning, focusing on architecture design and end-to-end latency analysis. Furthermore, they explored the vulnerability detection outcomes.

## 3 Federated learning framework with non-IID data based on blockchain

Federated learning, a form of distributed machine learning, often involves heterogeneous devices within the federation framework, leading to heterogeneity in local data distribution. In the process of training local models from an initial global model received by local devices, the slow convergence or even non-convergence of local models is due to non-IID data on local devices resulting in poor performance of traditional federated learning. Therefore, investigating federated learning on non-IID data is worthy of further exploration. Diverging from the conventional federated learning framework, our proposition involves constructing a data-sharing layer based on blockchain technology to establish a globally shared dataset by clients sharing small portions of their local data. Before federated learning training begins, clients need to download the globally shared dataset, train on it together with their local data, and perform local aggregation to provide the latest local model to the server, which can enhance the performance of federated learning and ensure the proper functioning of the global model.

Furthermore, although federated learning adopts a distributed training method that significantly safeguards the privacy and security of local data by aggregating models globally after local training, there are still many potential privacy threats. Malicious attacks from other devices can be categorized into two distinct types: attacks against model data and attacks against the model itself. For example, malicious adversaries can harm client users by poisoning original data [40] and affecting the model training results, or use gradient inversion attacks to counteract model updates and pose threats to the security and privacy protection of federated learning [41]. To address this, we utilize smart contracts to issue initial models and receive locally aggregated models, to realize the decentralized process of federated learning, protect model security, and improve learning performance.

### 3.1 Architecture of a federated learning system based on hierarchical blockchain

**Figure 2:**
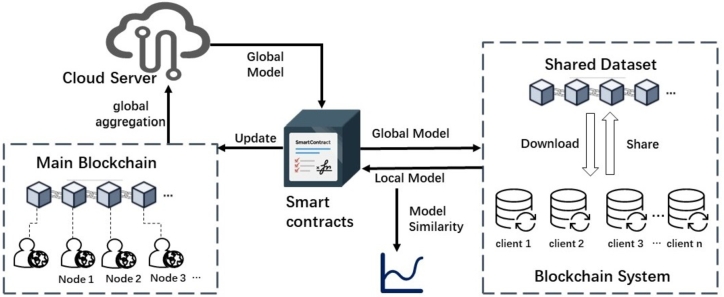
The Architecture of a Federated Learning System Based on Hierarchical Blockchain.

A hierarchical blockchain for federated learning on non-IID data is presented in Fig. 2, where a blockchain-based system at the data-sharing layer constructs a globally shared dataset for local model training and aggregation. In addition, a main blockchain is employed to facilitate global model aggregation across all users.

We report the hyperparameters of our experiments in Table 1.

**Table 1:** Symbolic representations used in our framework.

Symbols	Meaning
*t*	Federated Learning Rounds.
*M*_0_	Initial model.
*M*_*n*_	Local model.
*M*_*g*_	Global model.
*A*_*α*_	Local shared data.
*A*_*i*_	Local data and shared data.
*A*_*g*_	Global shared dataset.
*ω*^0^	Original model parameter configuration.
*ω*_*t*_	Model parameter configuration at epoch t.
$\omega_{t}^{i}$	Local model parameter configuration at epoch t.
*F*_*i*_()	Loss function.
*p*_*j*_	Ratio of local data and shared data to the total number of samples.
*η*	Learning rate.

### 3.2 Smart contacts

The smart contract is an automated computer program built on blockchain technology, capable of executing tasks automatically and controlling various operations and behaviors specified in the contract terms. Smart contracts are often considered one of the important applications of blockchain technology, as they can achieve decentralization, automation, and trust mechanisms, and play an important role in multi-party collaboration and transactions. The proposed utilization of smart contracts in this paper is founded on the Block FL architecture presented in [34] for horizontal federated learning, with certain enhancements incorporated. The operational steps of the smart contract proposed in this paper are as follows:

Step 1: The authority sets the current round e of federated learning and the initial model M0. The smart contract issues the initial model M0 to the clients, who train the initial model *M*0 with their local data to obtain the local model Mn.

Step 2: Each client uploads the Mn to the smart contract, which performs workload proof for the model accuracy based on its own test set and issues an on-chain permit. If the time for one round of federated learning is up or enough local models have been collected, the smart contract sends an aggregation signal to the server.

Step 3: The server retrieves the queue of local models for a round of federated learning from the blockchain, applies the Federated Averaging algorithm to aggregate the local models, obtains a global model, and subsequently stores it in the main blockchain.

Step 4: The smart contract sends a signal to the clients to start the next round of decentralized federated learning using the aggregated global model.

Compared with the original paper, the above strategy replaces miner verification with smart contract verification and uses the Raft algorithm [8] for blockchain node consensus, while retaining the decentralization concept and mechanism.

### 3.3 Local model aggregation

In the conventional federated learning framework, the central server distributes initial models and initialization parameters to all clients at the commencement of the training process., which are then used by the clients to train local models with their local data. The traditional framework performs well on training with identically distributed data, but its performance declines significantly when considering non-IID data, resulting in relatively poor training results. To enhance the performance of federated learning on non-IID data, this paper introduces a blockchain-based data-sharing strategy aimed at reducing the heterogeneity of local client data. A uniformly distributed data-sharing set is constructed as part of this approach, where data comes from partial local data of each client, and this dataset into a new local dataset to avoid insufficient training of initial models.

Algorithm 1 outlines the general workflow of the proposed architecture. In our proposed architecture, small-scale global shared data is set up via the blockchain system prior to the federated learning process. The main workflow is summarized as follows:

**Algorithm 1:**
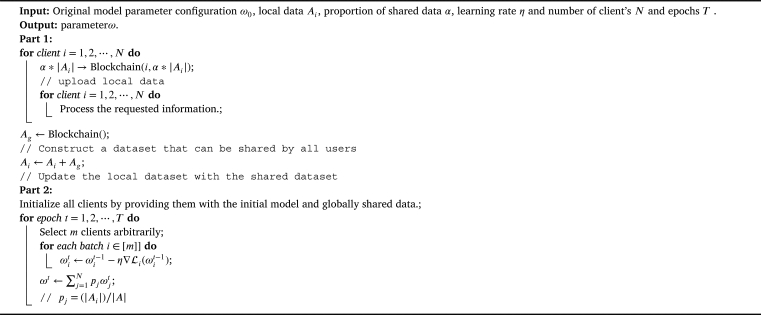
Local Model Aggregation.

Step 1: Client identification by the blockchain system. The client calculates a hash value through a workload-proof consensus mechanism and sends a shared data request to the blockchain system. After the verification process is passed, the blockchain is initialized.

Step 2: The client contributes a portion of its local data to the blockchain system, resulting in the generation of a global shared dataset.

Step 3: The client incorporates the shared dataset into its local data, leading to a modification of the loss function as follows:

(1) $$F_{i}\;(\omega^{i}\;)=l(D_{i},\omega^{i})\;\text{where}\;D_{i}=D_{i}+D_{g};$$

The size of the shared dataset $\left|D_{g}\right|$ is $\sum_{i}^{n}\alpha •|D_{i}|$. The updated global federated learning optimization objective is:

(2) $$\underset{\omega_{t}}{\mathrm{argmin}}\sum_{i=1}^{m}p_{j}l\left(D_{i}+D_{g},\omega^{i}\right);$$

Step 4: The client trains its local federated learning model with the updated local data and uploads the training result to the smart contract. The smart contract updates the main blockchain.

Step 5: Based on the main blockchain, the server conducts global aggregation to generate a new global model. Once the clients complete the *t*th round of training, they upload their gradients to the server. The parameters of the global model for the t+1th round are then updated according to the following procedure:

(3) $$\omega_{t+1}\leftarrow \omega_{t}-\eta \sum_{i=1}^{m}p_{j}\nabla F_{i}(\omega_{t}^{i});$$

Step 6: The smart contract distributes the latest global model to client users for their participation in the new round of training. If the model reaches the expected accuracy or the specified training cycle is completed, the process ends; otherwise, go back to step 1.

### 3.4 Model similarity

Model similarity is a metric used to compare the similarity between two or more machine learning models, typically calculated using some form of distance or similarity measure [42]. In model similarity calculations, differences between models can be compared in terms of their parameters, structures, outputs, and other aspects. Model similarity calculations can be used in fields such as model selection, model fusion, and model transfer learning, and can also be used to evaluate the robustness and stability of models [43]. Common methods for calculating model similarity in deep learning include Euclidean distance, cosine similarity, Manhattan distance, and others. In references [8], Zhao et al. proposed and derived the weight difference of models to address the impact of non-IID data on the Federated Averaging aggregation algorithm, as follows:

(4) $$weight\;divergence=\frac{∥W^{FedAvg}-W^{SGD}∥}{∥W^{SGD}∥};$$

Based on this work, we amplify the parameter differences between each layer, and sum up the weight differences between each layer as the model similarity between the two models, calculated as follows:

(5) $$S_{M_{n}M_{0}}=\sum_{k=1}^{n}\frac{{\left(∥P_{M_{n_{k}}}-P_{M_{0_{k}}}∥\right)}^{2}}{∥P_{M_{0_{k}}}∥};$$

To assess the effectiveness of the model similarity calculation method, we compute the variance of the model similarity between the local models and the initial model in each round of federated learning. This allows us to observe the extent of dissimilarity between the two models. The variance is calculated using the following formula:

(6) $$\sigma^{2}=\frac{\sum_{i=1}^{n}{\left(s_{i}-\overset{¯}{s}\right)}^{2}}{n};$$

### 3.5 Global model aggregation

Upon completion of client-side training, the locally trained model is recommended to take part in the rejuvenation of the global model. To report the local model to the main blockchain, a smart contract creates a new transaction and broadcasts the local model to the main blockchain through chain nodes for global aggregation. A dedicated chain node in the main blockchain oversees the global model aggregation by combining the locally trained models recorded in the main blockchain. Furthermore, a specialized chain node can be established within the main blockchain to record the global model for other users. Consequently, the global model undergoes updates solely through federated averaging at each epoch t of D samples, as outlined below:

(7) $$\omega_{t+1}\leftarrow \sum_{i\in N}\frac{D_{i}}{\left|D\right|}\omega_{t}^{i};$$

## 4 Experiment

In this chapter, we will conduct an experimental analysis of the proposed federated learning framework, and compare the performance improvement of decentralized federated learning strategies under non-IID data through comparative experiments. We will discuss the experimental environment setup, experimental settings, comparative experiments, and experimental analysis in four aspects.

### 4.1 Experimental environment

The experimental environment consists of a GPU server with one Quadro P5000 GPU (16 GB RAM) and CentOS 7 operating system. Machine learning functions are programmed using the PyTorch framework based on Python 3.7. The back-end programming uses the Gin framework and the smart contract of the Hyperledger fabric is written in Golang. For the experiment, we used version 1.4 of Hyperledger fabric and divided the peer nodes into three organizations, each with two peer nodes. Each organization deployed a smart contract on one node and enabled five order nodes to execute the Raft consensus service. In federated learning, a single model is trained on a GPU server and uploaded to one of the smart contract nodes.

### 4.2 Experimental setup

In this chapter, we select three common datasets to achieve our goal of classification. The first training task is to classify the MNIST dataset using a Multilayer Perceptron (MLP) model. During training, the MLP model updates the weights and biases between neurons through the backpropagation algorithm to minimize the loss function. After training, the data is classified. The second training task is to classify the Fashion-MNIST image dataset using a Convolutional Neural Network (CNN) model. CNN generates convolutional feature maps using 3x3 convolutional kernels to extract different features. ReLU functions activate two convolutional layers, followed by linear and softmax layers for output. Similarly, in the third training task, we use a CNN model consisting of three convolutional layers to train the CIFAR-10 dataset. The training and test sample information for the three training sets is shown in Table 2. In the three training tasks, we presume ten clients engage in model training. Each user arbitrarily selects data from the training set to create their local dataset.

**Table 2:** Datasets.

Dataset	Training samples	Test Samples	Classes	Model
MNIST	60,000	10,000	10	MLP
Fashion-MNIST	60,000	10,000	10	CNN
CIFAR-10	50,000	10,000	10	CNN

We reported our experimental parameters and corresponding performance metrics as follows:

• *α*: The shared data ratio. The value ranges from 0 to 1. When *α* equals 1, it indicates that the user shares all local data; when *α* equals 0, it indicates that the user does not share any data.
• *β*: Non-IID degree. The value ranges from 0 to 1. When *β* equals 1, it indicates that the data does not contain any additional categories of data; when *β* equals 0, it signifies that the data exclusively pertains to the same category.
• *σ*: The variance of model similarity. A larger variance of model similarity indicates greater performance differences among multiple models, and vice versa.
• *acc*: Model's accuracy on testing samples.

### 4.3 Analysis

#### 4.3.1 Model accuracy

In our training task, we investigated the impact of different shared data ratios and non-IID degrees on model accuracy. We took three different values of non-IID degree, namely *β* = 0.2, *β*= 0.6, *β* = 1, representing the proportion of different categories of data, and designed three sets of comparative experiments. Each set of experiments used the same non-IID degree and different shared data ratios for three different training tasks to explore the change in model accuracy under different model parameters.

**Figure 3:**
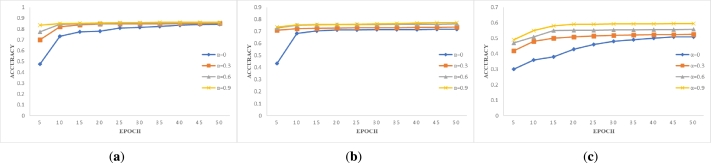
Accuracy versus epoch on *β*=0.2. (**a**) MNIST; (**b**) Fashion-MNIST; (**c**) CIFAR-10.

**Figure 4:**
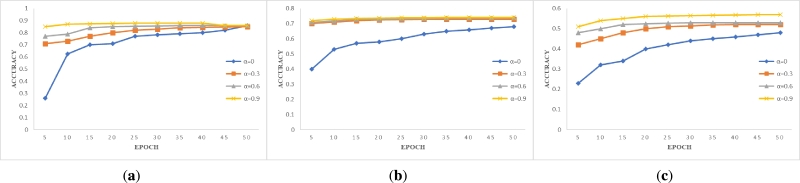
Accuracy versus epoch on *β*=0.6. (**a**) MNIST; (**b**) Fashion-MNIST; (**c**) CIFAR-10.

**Figure 5:**
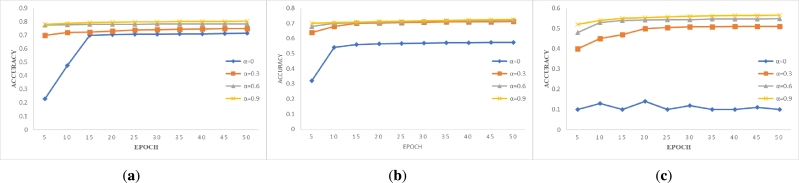
Accuracy versus epoch on *β*=1. (**a**) MNIST; (**b**) Fashion-MNIST; (**c**) CIFAR-10.

The results of training all data under different parameters are shown in Figure 3, Figure 4, Figure 5. Through the intuitive demonstration of nine subgraphs, we can conclude that non-IID degree has a significant impact on model accuracy. The experimental results align with the real-world scenario of federated learning when training with non-IID data. When we do not use the blockchain system designed by us for data sharing, i.e. α=0, we find that the accuracy of the MNIST and Fashion-MNIST datasets can still be improved with the increase of epoch, even under different non-IID degrees in Fig. 3 and Fig. 4. However, when the non-IID degree is high and training is performed on a more complex dataset such as CIFAR-10, the model may not converge, and the accuracy hovers around 0.1, which is comparable to random selection in Fig. 5. However, when we use our designed blockchain system to share data locally, all models converge. By observing the changes in the curves depicted in the graph, we can discover the advantages of our framework. With the continuous increase of *α* value, i.e. the more data is shared, the higher the model accuracy will be, and the model accuracy increases rapidly when t < 20, but the improvement rate tends to be flat when t > 20. Therefore, by sharing data, higher model accuracy can be obtained in fewer epochs. Through the comparison of the training results of the same dataset and non-IID degree, we found that only a small amount of data sharing can achieve high returns. Although continuously sharing data can indeed improve model accuracy, the improvement rate is constantly decreasing. Moreover, a small amount of data sharing can achieve good training results, which not only saves costs but also does not cause serious privacy leakage and data security issues for clients. The experimental results presented above validate the feasibility and effectiveness of the system we have designed.

#### 4.3.2 Model similarity

**Figure 6:**
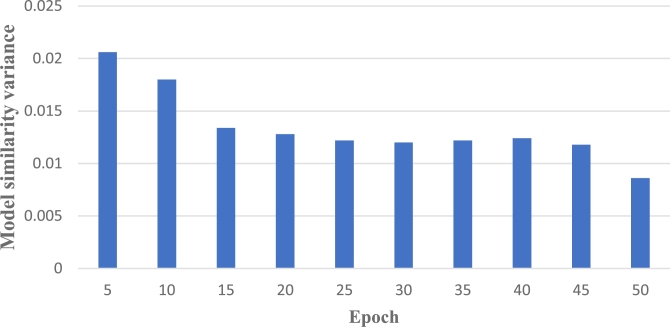
Fashion-MNIST's model similarity variance.

**Figure 7:**
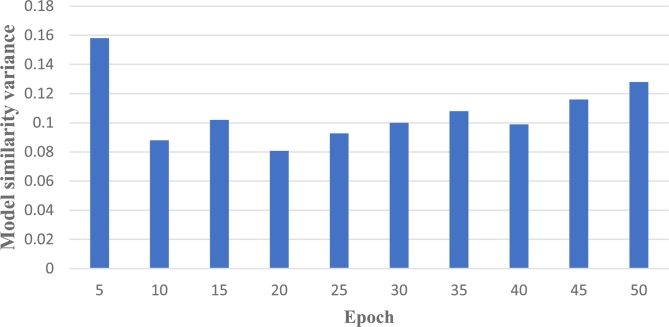
CIFAR-10's model similarity variance.

In this section, we calculate the model similarity of two difficult-to-train datasets Fashion-MNIST and CIFAR-10, and conduct a comparative experiment. The variance of model similarity between the two models at different epochs is shown in Fig. 6 and Fig. 7. Observing the two graphs, when performing federated learning on the same non-independent and identically distributed dataset, at the initial stage, significant differences exist among the local models due to variations in individual local data, resulting in a high variance in the corresponding model similarity. However, with the increase of rounds and several model aggregations, the differences between local models become smaller and the variance of model similarity tends to be relatively stable. This indicates that the performance difference between the trained local models and the initial models is small, and the globally aggregated model obtained through aggregation will be more representative.

#### 4.3.3 Number of clients

To magnify the impact of shared data amount and non-IID degree on model accuracy, we set the same number of clients in the previous section. This section explores the impact of the number of clients on training results. We selected the representative dataset CIFAR-10, which is the most difficult to train, and selected the better experimental parameters during training, assuming that each client has 1000 data and the shared data amount and non-IID degree are both 0.6. We conducted tests on the accuracy after 50 epochs, considering only the number of clients as a variable. The number of clients was set to 1, 5, 10, 15, and 20. The training results are presented in Table 3.

**Table 3:** Accuracy as a function of the client's number.

Number of clients	1	5	10	15	20
Accuracy	0.292	0.488	0.569	0.614	0.655

Fig. 7 visually demonstrates that the accuracy rises as the number of clients increases. Nevertheless, the rate of accuracy improvement gradually diminishes. The most substantial enhancement in accuracy occurs when transitioning from a single client to multiple clients. This aligns with our expectations that in a federated learning system, the involvement of diverse participants results in a larger pool of training data, which is highly beneficial for model training.

By examining Table 3, we can gain a clear understanding that as the number of clients increases, the model's accuracy improves continuously, albeit at a decreasing rate. The increase in the number of clients leads to the generation of more training data, and the greater diversity in data proves advantageous for model training. This observation aligns with the real-world scenario of a federated learning system.

## 5 Conclusions and future work

To address the challenge posed by non-IID data in federated learning, this paper introduces a hierarchical federated learning framework that leverages blockchain technology. The data-sharing layer collects local partial data integrates them into a global data-sharing set, and executes a blockchain-based data-sharing strategy. The main blockchain layer collects local models through smart contracts, stores them in chain nodes, and performs global aggregation to optimize the federated training process. Finally, Comparative experiments were conducted on three datasets, and the results indicate that even sharing a small amount of data can enhance system accuracy in non-IID data training. Moreover, the performance of decentralized federated learning using this strategy exhibits significant improvements across various tasks. As for future research directions, we will focus on addressing the issue of backdoor attacks in federated learning. To the best of our knowledge, backdoor attacks under non-IID data establish strong connections between backdoor samples and target labels, causing significant performance degradation in federated learning. This is an area that warrants in-depth investigation.

## CRediT authorship contribution statement

**Feng Zhang:** Writing – original draft, Methodology, Formal analysis, Conceptualization. **Yongjing Zhang:** Methodology, Data curation. **Shan Ji:** Validation, Software, Data curation. **Zhaoyang Han:** Visualization, Validation, Software.

## Declaration of Competing Interest

The authors declare that they have no known competing financial interests or personal relationships that could have appeared to influence the work reported in this paper.
